# Sarcomatoid hepatocellular carcinoma (SHC): a case report

**DOI:** 10.1186/s12957-017-1286-1

**Published:** 2017-12-12

**Authors:** Yingying Yu, Yanping Zhong, Jingyu Wang, Di Wu

**Affiliations:** 1 Department of Oncology, First Hospital of Jilin University, 71 Xinmin St., Changchun, Jilin 130012 China; 2 Department of Pathology, First Hospital of Jilin University, Changchun, Jilin 130012 China; 3 Department of Radiology, First Hospital of Jilin University, Changchun, Jilin 130012 China

**Keywords:** Sarcomatoid carcinoma, Hepatocellular carcinoma, Sarcomatoid hepatocellular carcinoma, Hepatitis C virus, Sarcomatoid transformation

## Abstract

**Background:**

Sarcomatoid hepatocellular carcinoma (SHC) is a rare malignant hepatic tumor. Recurrent interventional therapies such as transcatheter arterial chemo-embolization (TACE), radiofrequency ablation (RFA), and percutaneous ethanol injection have been reported previously utilized in a majority of SHC cases. To date, the exact pathogenic mechanisms underlying sarcomatoid transformation of hepatocellular carcinoma (HCC) remain unknown.

**Case presentation:**

In this study, we report a 68-year-old female SHC patient admitted to our hospital due to discrete abdominal distention for more than 20 days. Abdominal computed tomography (CT) with tri-phase enhancement revealed portal vein tumor thrombi (PVTT) and a left hepatic lobe lesion measuring 110.0 mm × 160.0 mm. The patient subsequently underwent liver resection, after which pathological examination revealed proliferation of spindle-shaped SHC cells. A sarcomatoid, T4 stage carcinoma was eventually diagnosed. Forty-seven days after the operation, tri-phase enhanced CT detected extensive lesions in the liver, spleen, peritoneum, omentum majus, and mesentery, indicating SHC recurrence and metastases. Combination chemotherapy with pirarubicin and cisplatin was initiated for 1 cycle, but terminated due to resultant severe myelosuppression and medication intolerance. The patient was lost to therapy after 3 months of follow-up.

**Conclusions:**

This case is unique because of hepatitis C virus infection. We should consider the possibility of this disease in patients with atypical clinical presentation.

## Background

Sarcomatoid carcinoma, a relatively rare form of malignant tumor, is characterized by features of epithelial and mesenchymal tumors. It is reported in a wide range of organs, with the lungs being most susceptible [1–3]. Sarcomatoid hepatocellular carcinoma (SHC) is a rare neoplasm of the liver, accounting for 1.8–2.0% of all patients undergoing surgery [4, 5]. The diagnosis of SHC requires postoperative pathological examination, while the preferred therapy is surgical excision. The prognosis of SHC remains very poor due to frequent recurrence and metastasis.

Clinical studies have suggested recurrent interventional therapies such as transcatheter arterial chemoembolization (TACE), radiofrequency ablation (RFA), and percutaneous ethanol injection to potentially be major causes of sarcomatoid transformation of hepatocellular carcinoma (HCC) [6–8]; only a few cases were reported in SHC patients without receiving the above therapy [9]. To date, the precise etiology of SHC remains unclear.

In this case report, we describe a patient previously diagnosed with hepatitis C on discontinuous antiviral therapy manifesting with SHC. Additionally, we review the histopathologic, immunohistochemical, and diagnostic characteristics of SHC.

## Case presentation

**Fig. 1:**
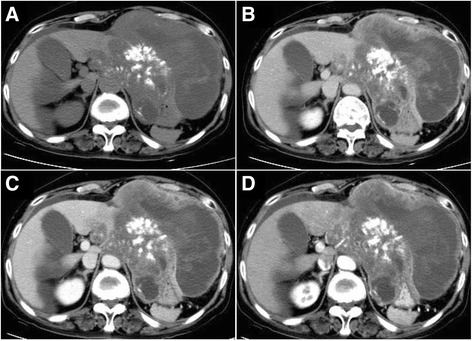
Abdominal CT images prior to surgical excision. **a** An oversized mass shadow of mixed density extrudes towards the front of the left hepatic lobe. An irregular cystic portion can be observed with scattered patch- and nodal-like calcification. **b**–**d** The signal of lesion tissue was not obvious under enhanced scan, while a contrast scan reveals a substantial portion to be enhanced. A mass-like shadow with relatively low density is noted in the internal area of the neighboring left hepatic lobe. On enhanced CT, the margins of arterial and delayed phases are significantly enhanced, whereas the equilibrium phase is unclear. The boundary of the neighboring anterior gastric wall and lesser curvature is unclear. A strip-like low-density filling defect is observed in the left portal vein, which is unclear distally

A 68-year-old female patient was admitted to our hospital due to perceived abdominal distention for over 20 days. Past medical history included an 8-year infection with HCV and discontinuous anti-viral treatment. Laboratory tests revealed levels of HCV antibodies of 32.900 S/CO (< 1.00), carbohydrate antigen 125 of 361.69 U/ml (< 35.00), neuron-specific enolase of 62.05 ng/ml (< 25.00), and alpha- fetoprotein (AFP) of 113.77 ng/ml (< 20.00). Abdominal computed tomography (CT) scan with tri-phase enhancement revealed an irregular, dense shadow (dimensions 110.0 mm × 160.0 mm) in the left hepatic lobe with cystic fibrosis and scattered uneven calcification within. An abnormally low-density filling defect was observed in the left portal vein (Fig. 1a–d).

The patient was scheduled to undergo left hepatic resection, during which an oversized but uncoated mass was detected. The tumor had invaded into the adipose tissue external to the hepatic capsule and grown in with tumor thrombi in the portal vein. Tumor invasion was not observed into nerve and incisal edge tissue, while the surrounding hepatic tissue was obviously infected with chronic hepatitis (G2S2).

**Fig. 2:**
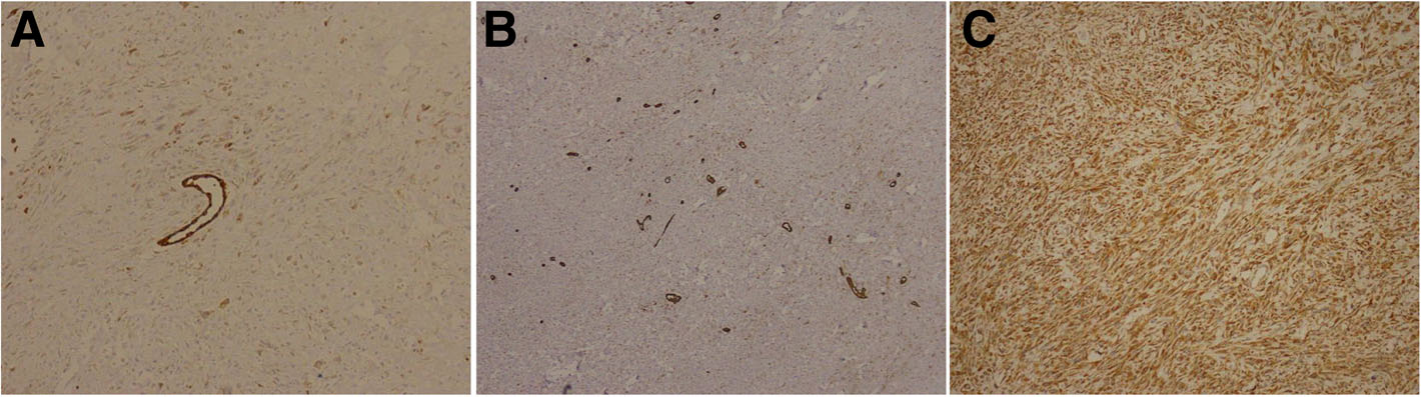
Immunohistochemical examination of a tumor segment (×400). **a** Partial positivity for cytokeratin (CK). **b** Partial positivity for cytokeratin-8 (CK-8). **c** Positivity for vimentin

**Fig. 3:**
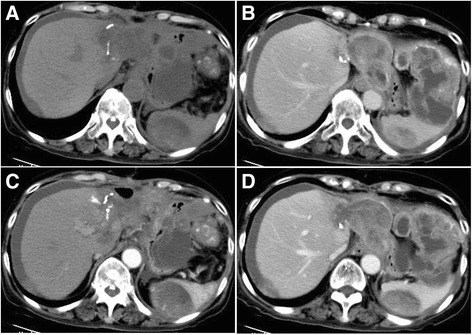
Abdominal CT imaging 47 days after surgical excision. **a**, **d** The outer area of the left hepatic lobe is absent. Extensive massive, nodal-like shadows can be found in the liver, spleen, peritoneum, omentum majus, and mesentery. **b**, **c** Heterogeneous enhancement is noted. There is no clear boundary between the lesion, the incisal edge of the left hepatic lobe, and the gastric wall. A low-density filling defect can be observed in the main portal vein. A flake-like shadow of liquid density is observed on the surface of the liver and in the inter-intestinal space

**Fig. 4:**
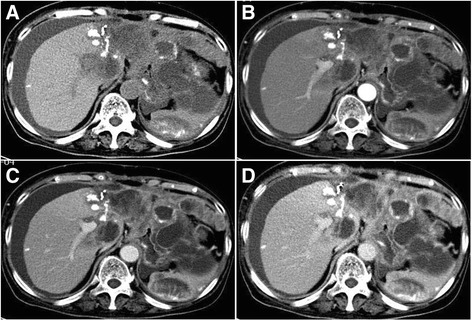
Abdominal CT imaging after chemotherapy. **a** The left hepatic lobe is absent. Space-occupying lesions are seen throughout the liver, spleen, peritoneum, omentum majus, and mesentery. **b**–**d** Extensive massive, nodal-like shadows with heterogeneous enhancement are seen in the liver parenchyma, spleen, peritoneum, omentum majus, and mesentery. There was no clear boundary between the lesion, the incisal edge of left hepatic lobe, and the gastric wall. A low-density filling defect can be observed in the main portal vein

Immunohistochemistry revealed carcinoma cells positive for CD34 and CK8 (focalin). Cells were also partially positive for smooth muscle actin (SMA), vimentin, and cytokeratin-pan (CK-pan), but negative for desmin and AFP (Fig. 2a–c). Histology revealed proliferation of spindle-shaped SHC cells; the cancer was staged at T4. Forty-seven days after the operation, tri-phase enhanced CT detected extensive massive, nodal-like regions in the liver, spleen, peritoneum, omentum majus, and mesentery. These heterogeneous densities with unclear boundaries at the incisal edge of the left hepatic lobe and gastric wall suggested reoccurrence and metastases of the SHC. A low-density filling defect was noted in the main portal vein (Fig. 3a–d). Chemotherapy was conducted with pirarubicin and cisplatin for 1 cycle; the curative effect was evaluated as stable disease (SD) (Fig. 4a–d), and the patient was followed up for 3 months before quitting therapy.

## Discussion

SHC is a rare form of a malignant hepatic tumor with combined characteristics of both HCC and hepatic sarcoma. In this case report, we described a unique case of an SHC patient previously diagnosed with HCV infection not having received anticancer therapies. We reviewed associated pathological and imaging features as well as relevant literature.

As in primary hepatic carcinoma, symptoms of SHC are non-specific, which may simply result in a diagnosis of HCC at an advanced stage. However, in contrast to patients with HCC, those suffering SHC are more likely to develop local recurrence and distant metastases after surgical excision. In the present case, laboratory tests indicated abnormally high levels of AFP, which declined and fell within the normal range after surgery, suggesting that AFP may be associated with sarcomatoid transformation. Imaging may provide information assisting localization of the lesion. Currently, surgical excision is the preferred effective treatment for SHC, as the efficacy of alternative treatments such as radiotherapy, chemotherapy, and targeted therapy remain unclear.

### Pathological features and pathogenic considerations

**Fig. 5:**
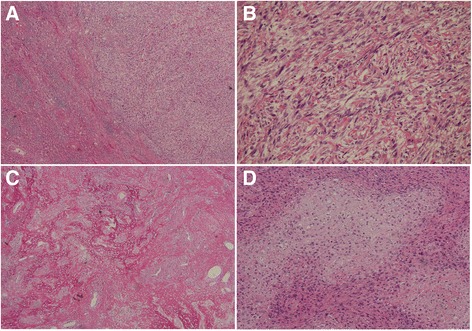
Pathological findings of a tumor segment. **a** Lesion tissue in liver (HE × 10). **b** Flaky- or bunchy-disposed cells characterized by spindle-shaped SHC cells, clear nucleoli, acidophilic cytoplasm, atypia, and significant mitotic figures (HE × 40). **c**, **d** Bone and cartilage differentiation (HE × 20)

Final diagnosis of SHC depends upon precise histopathological, immunohistochemical, and pathological techniques. The primary hepatic sarcoma cell is histologically characterized by a spindle shape, possessing a clear nucleolus and acidophilic cytoplasm, atypia, and significant mitotic attributes [5]. Sarcomatous epithelium is composed of adenocarcinoma cells, which have a hyperchromatic nucleolus and mitotic attributes. Heterologous sarcomas can be detected in some cases, such as in cancerous bone and cartilage components, as we report (Fig. 5a–d). The microscopic characteristics of SHC in our case are consistent with previous studies. Although immunohistochemical examination for epithelial and mesenchymal markers is helpful in diagnosing SHC, specific biomarkers for the disease have not been confirmed to date. In general, SHC lesions are positive for both an epithelial marker (cytokeratin) and a mesenchymal marker (vimentin). In this study, we detected positivity for cytokeratin, epithelial membrane antigen (EMA), CD34, SMA, and vimentin. A diagnosis of SHC was made based on these findings, and the pathological characteristics of the lesion were in agreement with those of previous SHC studies.

The pathogenesis underlying SHC remains unclear. Most reported SHC cases manifested in the elderly patients who previously received recurrent interventional therapies such as TACE, RFA, or percutaneous ethanol injection [6–8]. A number of previous reports indicated that sarcomatous appearance was highly prevalent in HCC patients who have undertaken various anti-cancer treatments, including TACE, RFA, or percutaneous ethanol injection, suggesting that recurrent therapies for HCC are likely to promote sarcomatoid transformation of the cancer [6–8]. However, sarcomatoid transformation of HCC or SHC was also reported, although extremely rare, to occur in cases who had not received any previous recurrent interventional therapies including the present case. It was of note that our patient was detected positive for HCV, a RNA virus that has been well-recognized to contribute to hepatocellular carcinogenesis. It has been shown that various forms of HCV-associated liver cancer including HCC, hepatocholangiocarcinoma, and cholangiocarcinoma were histologically different from those negative for HCV [10, 11]. Some of HCV-associated neoplasms suggested sarcomatous changes and epithelial-mesenchymal transition [12–14]. Thus, there is a possibility that HCV infection could play a part in the development of SHC in the present case.

### Imaging features

Imaging remains the primary modality used in localization of lesions. It also provides essential information in establishing an accurate diagnosis, which is obviously vital for choosing and optimizing the type of surgery for patients with SHC. The rapid progression of SHC usually results in a massive, cystic-solid mass of mixed density noted on imaging exhibiting characteristic CT findings. A lower-density shadow in the mass is frequently detected, likely caused by ischemia and necrosis of the tumor due to rapid cellular proliferation. On enhanced CT scan, central mass signal intensity decreases due to necrosis, while parenchymal signal intensity remains greater. Similar enhancements can be found in the margins of the arterial and delayed phases. SHC exhibits weak signal intensity on T1W1 MRI, but high signal intensity on T2W1. On enhanced MRI, a substantial margin of the irregular septum is enhanced during the arterial phase, while the central cystic necrosis does not, and the portal venous phase reveals decreased enhancement [15]. In our patient, abdominal CT revealed an irregular dense shadow measuring 110.0 mm × 160.0 mm in the left hepatic lobe and an abnormal low-density filling defect in the left portal vein. These findings suggested the presence of a poorly differentiated liver cancer with PVTT.

### Antidiastole of SHC

Literature regarding SHC mostly consists of case reports, due to the low incidence of the condition and unconfirmed diagnostic criteria. It is important that SHC is differentiated from other liver tumors, such as hepatapostema and hepatosarcoma. Common clinical symptoms of hepatapostema include pain in the right upper abdominal quadrant, fever, and chills. Laboratory tests usually indicate the occurrence of an inflammatory reaction manifesting with anemia, increased white blood cell count, elevated erythrocyte sedimentation rate, and higher expression of C-reactive protein. Imaging of hepatapostema typically reveals an isolated mass without liver lobe infiltration. Pathological examination exhibits phlogotic infiltration into the lesion instead of sarcomatous and carcinomatous constitution [16]. Hepatosarcomas contain true epithelial and mesenchymal components, a finding key to differentiating them from sarcomatoid carcinomas [16]. Epithelial components include both adenocarcinomatous and squamous carcinomatous cells, in which CK and other epithelial biomarkers are positively expressed. Mesenchymal components include osteosarcomatous, chondrosarcomatous, fibrosarcomatous, and rhabdomyosarcomatous cells, in which vimentin and other mesenchymal biomarkers can be detected. Imaging may suggest calcification and bone formation, regarded as additional points of differentiation from sarcomatoid carcinoma.

### Treatment and prognosis

Surgical excision is accepted as the most effective treatment of SHC, but patients are more likely than those with typical HCC to develop local recurrence and distant metastases after operation, mainly due to the high grade of malignancy and rapid progression of the disease. Indeed, the prognosis of SHC is quite poor, with 3-year survival rates reported as low as 18.2% after hepatectomy [17]. To date, the effectiveness of alternative treatments such as radiotherapy, chemotherapy, and targeted therapy remain unclear. As observed in the majority of SHC cases, our patient experienced a relapse 47 days after the perceivably curative liver resection, with tumor recurrence and metastasis found in the residual liver, spleen, peritoneum, omentum majus, and mesentery, consistent with previous case reports.

## Conclusion

In summary, we report a rare case of SHC, in which HCV infection might have contributed to its sarcomatoid transformation. When treating atypical HCC patients with viral hepatitis, even those not undergoing anticancer therapies, SHC should be considered as a possible diagnosis. A poor prognosis, with extremely low 5-year survival rates, should be expected. Future studies are urgently needed for exploring more effective therapies in order to extend the survival period and improve the quality of life of patients with SHC.

## Abbreviations

AFP: Alpha-fetoprotein
CD34: Cluster of differentiation 34
CK8: Cytokeratin 8
CK-pan: Cytokeratin-pan
CT: Computed tomography
EMA: Epithelial membrane antigen
HCC: Hepatocellular carcinoma
HCV: Hepatitis C virus
MRI: Magnetic resonance imaging
PVTT: Portal vein tumor thrombi
RFA: Radiofrequency ablation
SMA: Smooth muscle actin
TACE: Transcatheter arterial chemo-embolization

## References

[1] Kojiro M, Sugihara S, Kakizoe S, Nakashima O, Kiyomatsu K (1989). Hepatocellular carcinoma with sarcomatous change: a special reference to the relationship with anticancer therapy. Cancer Chemother Pharmacol.

[2] Nonnis R, Paliogiannis P, Giangrande D, Marras V, Trignano M (2012). Low-grade fibromatosis-like spindle cell metaplastic carcinoma of the breast: a case report and literature review. Clin Breast Cancer.

[3] Chrysikos D, Zagouri F, Sergentanis TN (2012). Mucinous tubular and spindle cell carcinoma of the kidney: a case report. Case Rep Oncol.

[4] Seok JY, Kim YB (2010). Sarcomatoid hepatocellular carcinoma. Korean J Hepatol.

[5] Giunchi F, Vasuri F, Baldin P (2013). Primary liver sarcomatous carcinoma: report of two cases and review of the literature. Pathol Res Pract.

[6] Marijon H, Dokmak S, Paradis V, Zappa M, Bieche I, Bouattour M, Raymond E, Faivre S (2011). Epithelial-to-mesenchymal transition and acquired resistance to sunitinib in a patient with hepatocellular carcinoma. J Hepatol.

[7] Kojiro M, Sugihara S, Kakizoe S, Nakashima O, Kiyomatsu K (1989). Hepatocellular carcinoma with sarcomatous change: a special reference to the relationship with anticancer therapy. Cancer Chemothe Pharmacolo.

[8] Obara K, Matsumoto N, Okamoto M, Kobayashi M, Ikeda H, Takahashi H (2008). Insufficient radiofrequency ablation therapy may induce further malignant transformation of hepatocellular carcinoma. Hepatol Int.

[9] Nakanishi C, Sato K, Ito Y (2012). Combined hepatocellular carcinoma and neuroendocrine carcinoma with sarcomatous change of the liver after transarterial chemoembolization. Hepatol Res.

[10] Jarnagin WR, Weber S, Tickoo SK, Koea JB, Obiekwe S, Fong Y, DeMatteo RP, Blumgart LH, Klimstra D (2002). Combined hepatocellular and cholangiocarcinoma: demographic, clinical, and prognostic factors. Cancer.

[11] Lee WS, Lee KW, Heo JS, Kim SJ, Choi SH, Kim YI, Joh JW (2005). Comparison of combined hepatocellular and cholangiocarcinoma with hepatocellular carcinoma and intrahepatic cholangiocarcinoma. Surg Today.

[12] Ishak KG, Anthony PP, Nicederau C, Nakanuma Y, Stanley RH, Lauri AA (2000). Mesenchymal tumours of the liver. WHO international histological classification of tumours, pathology and genetics of the tumours of the digestive system.

[13] Thiery JP (2002). Epithelial–mesenchymal transitions in tumour progression. Nat Rev Cancer.

[14] Battaglia S, Benzoubir N, Nobilet S, Charneau P, Samuel D, Zignego AL, Atfi A, Bréchot C, Bourgeade MF (2009). Liver cancer-derived hepatitis C virus core proteins shift TGF-beta responses from tumour suppression to epithelial-mesenchymal transition. PLoS One.

[15] Koo HR, Park MS, Kim MJ (2008). Radiological and clinical features of sarcomatoid hepatocellular carcinoma in 11 cases. J Comput Assist Tomogr.

[16] Amarapurkar AD, Vibhav, Kim V (2008). Angiogenesis in liver cirrhosis and hepatocellular carcinoma. Indian J Pathol Microbiolo.

[17] Hwang S, Lee SG, Lee YJ (2008). Prognostic impact of sarcomatous change of hepatocellular carcinoma in patients undergoing liver resection and liver transplantation. J Gastrointest Surg.

